# Does Juvenile Idiopathic Arthritis Affect the Course of Legg–Calvé–Perthes Disease? A Case-Control Study with a Mean Follow-Up of 8 Years

**DOI:** 10.3390/children8111014

**Published:** 2021-11-05

**Authors:** Julien Roß, Ivan Foeldvari, Kara L. Krajewski, Sebastian Butscheidt, Frank Timo Beil, Ralf Stücker, Alexander S. Spiro

**Affiliations:** 1 Department of Pediatric Orthopedics, Altonaer Children’s Hospital, Bleickenallee 38, D-22763 Hamburg, Germany; julien.ross@kinderkrankenhaus.net (J.R.); ralf.stuecker@kinderkrankenhaus.net (R.S.); 2 Department of Orthopedics, University Medical Center Hamburg-Eppendorf, Martinistr. 52, D-20246 Hamburg, Germany; s.butscheidt@uke.de (S.B.); t.beil@uke.de (F.T.B.); 3 Hamburg Center for Pediatric and Adolescence Rheumatology, Dehnhaide 120, D-22081 Hamburg, Germany; foeldvari@t-online.de; 4 Department of Pediatric Neurosurgery, Altonaer Children’s Hospital, Bleickenallee 38, D-22763 Hamburg, Germany; KaraLeigh.Krajewski@kinderkrankenhaus.net

**Keywords:** Perthes disease, juvenile idiopathic arthritis, pediatric orthopedics, pediatric rheumatology

## Abstract

Background: This study aimed to determine the clinical and radiological course in children who had Legg–Calvé–Perthes disease (LCPD) associated with juvenile idiopathic arthritis (JIA). Methods: In a retrospective chart review between 2007 and 2019, eight consecutive JIA patients diagnosed with concomitant LCPD were identified and compared with a case-control group of 10 children with LCPD only. Results: LCPD was diagnosed at a mean age of 8.1 years (3.0–14.7) in children with JIA as compared to 6.1 years (2.9–10.0) in controls. According to the modified Harris Hip Score (mHHS), four children with JIA and all controls had an excellent result. Regarding the fragmentation severity and the duration of each stage, we found no differences using the lateral pillar and modified Elizabethtown classification. Five hips were classified as Stulberg I/II, two hips as Stulberg III, and one hip as Stulberg V with no evidence of hip dysplasia or severe overcoverage in either group. Conclusions: The radiological outcome of LCPD did not differ between both groups, while the clinical outcome was slightly better in controls. Physicians should be aware that children with LCPD may have JIA too. In suspicious cases, further investigations are recommended, and patients should be referred to pediatric rheumatologists.

## 1. Introduction

Hip pain commonly presents as a limping child [1,2]. Legg–Calvé–Perthes disease (LCPD) and juvenile idiopathic arthritis (JIA) are potential differential diagnoses [3,4,5]. The etiologies of those disorders are not entirely understood [6,7,8]. Synovitis and joint effusion, which are suspected to play a significant role in the interruption of blood supply of the femoral head, occur in both disorders [9]. JIA with affected hip joints may result in femoral head necrosis as well [10,11]. The distinction between these two conditions can present a challenge in daily clinical practice. Symptoms on initial presentation may be non-specific.

A delayed diagnosis of LCPD or JIA may seriously affect joint congruency and mobility of the hip. Early diagnosis and treatment are therefore of utmost importance, considering that treatment and outcome still vary considerably in each case [12,13].

However, there has been no study published so far that reports on a series of LCPD patients with a verified diagnosis of JIA. In a review of the literature, we only found one report on two 6-year-old boys with unilateral LCPD who had oligoarticular juvenile chronic arthritis as well [9]. Both developed a contralateral femoral head necrosis after several years, leading to an ankylosis of the hip.

This study aimed to determine the clinical and radiological course and long-term outcome of Legg–Calvé–Perthes disease in children who had a concomitant juvenile idiopathic arthritis as compared to patients without this rheumatological condition. The authors hypothesized that JIA may affect the course and outcome of LCPD.

## 2. Materials and Methods

A retrospective chart review of all children with suspected diagnoses of JIA who were treated between 2007 and 2019 at our institution with confirmed LCPD was conducted. The electronic medical record system was searched for diagnosis-related groups, coding for pediatric rheumatological disorders and LCPD. In total, 38 medical charts of all patients with LCPD also included DRG codes for pediatric rheumatological disorders. A total of 8 of these patients with LCPD (7 boys, 1 girl) had JIA and were treated by I.F., being an acknowledged specialist in the field of pediatric rheumatology. To further prove that all patients with both diseases fit the International League of Associations for Rheumatology Classification of Juvenile Idiopathic Arthritis (2004) criteria for JIA, all charts were individually reviewed by one author (J.R.) and rechecked by the senior author (A.S.S.) [14]. Children without a confirmed diagnosis of JIA or Perthes were excluded. Missing clinical outcome data (mHHS), radiographs without signs of new bone formation at the periphery of the necrotic fragment, and a follow-up of less than two years were defined as exclusion criteria. To reach comparable long-term results, we matched these patients with ten children suffering from LCPD only (8 boys, 2 girls) with a similar gender and age distribution, as well as local origin and follow-up. The small sample size resulted from our focus on a complete clinical and radiological documented long-term outcome.

Demographic and clinical data such as gender, family history, age at diagnosis, time of initial symptoms, affected joints, leg length discrepancy, laboratory studies, imaging studies, treatment type, and range of motion of both hips over the course of treatment were recorded. Relevant clinical outpatient notes by the referring pediatric rheumatologist were reviewed to determine whether the patients met the criteria for active or inactive rheumatic disease status and what type of medication was used. Each patient’s medical record was also reviewed for body mass index (BMI) at diagnosis and at the last follow-up. BMI categories were determined from percentiles by using the Centers for Disease Control and Prevention pediatric BMI calculator with the following cutoffs: normal weight (5th to 84th), overweight (85th to 94th) and obesity (95th to 100th) [15].

The course of LCPD and the final clinical and radiographic outcomes were assessed in all patients. To evaluate the stage of evolution of LCPD, a modification of the original Elizabethtown classification published by Joseph et al., based on anteroposterior and frog-leg lateral projections, was used [16]. They modified the original Elizabethtown classification of Canale by further dividing the first three stages into substages as stage Ia, Ib, IIa, IIb, IIIa, IIIb, and IV. The duration of each stage was determined as the interval between the first radiograph showing features of one stage and the first radiograph showing features of the next stage. With regard to fragmentation severity, the original lateral pillar classification published by Herring et al. was applied, which has been proven to be both reproducible and of prognostic value [17,18,19,20]. At the latest follow-up, the affected hips were classified according to the Stulberg classification to predict long-term prognosis. With regard to the femoral head, a spherical congruency (groups I and II) was considered as a “good”, an aspherical congruency (groups III and IV) as a “fair” and an aspherical incongruency (group V) as a “poor” result [21]. A senior pediatric orthopedic surgeon (A.S.S.) and an orthopedic resident (J.R.) assessed the different classifications in a consensus-building meeting. On radiographs taken at the last follow-up, radiologic measurements were made in both the affected as well as in the unaffected hips with specific reference to the extrusion index, Sharp’s angle, lateral and medial center-edge angle, acetabular arc, and acetabular index [22]. The radiologic measurements were all performed by a single author (J.R.) and verified by the senior author (A.S.S.). The modified Harris Hip Score (mHHS) including eight questions and covering three domains (pain, function, and activities of daily living (ADL)) was used as a clinical outcome measure. Results of the mHHS were categorized as “excellent” (≥90 points), “good” (≥80 points), “fair” (≥70 points), or “poor” (≤70 points) [23].

Data were analyzed by using the SPSS statistical package. A comparison of the two different groups was performed either using the Fisher’s exact test or the non-parametric Mann–Whitney U test. Descriptive statistics were reported as number and percentage (%), mean, median, and standard deviation (SD), as well as range, as appropriate. Differences were considered statistically significant if the *p*-value was <0.05.

Ethics approval for the study was obtained from the local institutional review board (Reference number: WF-102/20).

## 3. Results

The mean age at the time of LCPD diagnosis was 8.1 years (3.0–14.7, median: 7.9) in the JIA group as compared to 6.1 years (2.9 to 10.0, median: 6.1; *p* = 0.315) in controls (Table 1 and Table 2). Most of these patients had juvenile idiopathic oligoarticular arthritis (five cases), followed by juvenile idiopathic enthesitis-related arthritis (two cases) and juvenile idiopathic psoriatic arthritis (one case). The mean age at the time of JIA diagnosis was 9.1 years (4.6 to 14.6, median: 9.0). LCPD had been diagnosed prior to JIA in four cases.

The average follow-up (FU) was 9.2 years (2.8 to 18.3, median: 9.2) in the JIA group and 7.1 years (3.3 to 14.4, median: 6.0; *p* = 0.515) in controls. None of the patients had bilateral LCPD. The left hip was involved in six cases in each group. A history of trauma was present in a small number of patients (1/8 in the JIA group and 2/10 in controls). Most of the JIA patients (5/8) had a positive family history of rheumatic or chronic inflammatory disease as compared to the control group, in which the family members were rarely affected by rheumatic or chronic inflammatory conditions (2/10; *p* = 0.088). The most common complaints of the JIA patients on initial presentation were hip pain (100%, 8/8), followed by limping (87.5%, 7/8), knee pain (50%, 4/8), and leg pain (12.5%, 1/8) on the side affected by LCPD. Most of the control patients had a limp at initial examination (80%, 8/10). Hence, hip pain was the second most common complaint (60%, 6/10), followed by knee pain (50%, 5/10) and leg pain (20%, 2/10) in this group. The BMI distribution was significantly different in the JIA group as compared to controls at the time of LCPD diagnosis and at the last follow-up (Table 3). Neither uveitis nor vasculitis were diagnosed by local ophthalmologists in the JIA group. None of the patients in the control group showed signs of uveitis or vasculitis.

Elevated inflammatory or rheumatic markers were observed in five patients in the JIA group (5/8; 1× HLA B27, 1× rheumatoid factor, 2× antinuclear antibodies (ANA), 1× increased blood sedimentation rate), but only in one patient of the control group (1/6; 1× ANA). Vitamin D deficiency was found in most of the JIA patients (6/8), while controls were rarely affected (1/10; *p* = 0.009).

Morning stiffness was reported by seven out of eight JIA patients (87.5%) during the course of disease as compared to four out of ten patients in the control group (40%; *p* = 0.057). Effusion of the affected hip joint (LCPD side) was seen in seven out of eight JIA patients and in all controls at initial presentation. Synovitis of the affected hip joint (LCPD side) was seen in five out of eight JIA patients but only in three out of ten controls. In addition, synovitis of the non-affected hip joint (not the LCPD side) was seen in two JIA patients but in none of the controls. The mean number of other joints affected was 5.1 (SD: 1.6, range: 3–7, median: 5.0) in the JIA group and 0.6 (SD: 0.7, range: 0–2, median: 0.5; *p* = 0.000) in controls until the last follow-up (*p* = 0.001). Knee (5/8), ankle (4/8), and wrist (5/8) complaints, as well as sacroiliitis (5/8), occurred frequently in addition to hip pain in JIA patients, but they were rarely seen in the control group (0/10 had ankle complaints, 0/10 had wrist complaints, and 0/10 had sacroiliitis (*p* = 0.007)); 2/10 had knee complaints (*p* = 0.184).

Nonsteroidal anti-inflammatory drugs (NSAIDs), including ibuprofen, naproxen, and meloxicam, were used as first-line treatment in all patients with JIA. Six of these patients were treated with methotrexate. In addition, biologicals (adalimumab or etanercept) were used in two JIA patients. Side effects of the medications were seen in seven out of eight JIA patients. NSAIDs were commonly used for managing pain associated with LCPD in the control group. However, naproxen was prescribed more frequently to children with JIA (*p* = 0.004).

Patients underwent a variety of treatments for LCPD in both groups (Table 1 and Table 2). All children except one in each group had surgery followed by physical therapy during the course of the disease. Conservative management included physical therapy, partial or non-weight-bearing with the use of crutches, and sport restriction. The most common surgical procedure was the proximal femoral varus osteotomy (4/8 in the JIA group versus 8/10 in controls). The amount of varus angulation was 20° in each JIA patient and 19.4° (SD: 5.6, range: 10–30, median: 20; *p* = 0.776) in the control group.

Based on the first available radiograph, five hips of the JIA group and six hips of the controls were classified as being in the late initial phase (Ib), two hips of the JIA group and three control hips were assigned to the early fragmentation phase (IIa), one control hip presented at the late fragmentation stage (IIb), and one hip of the JIA group was assigned to the reossification phase (IIIa). None of the patients who presented in the initial phase (Ib) bypassed the fragmentation stage completely. There were no significant differences concerning the duration of each stage between the two groups, although the transition time from stage II to IV was longer in JIA patients, with a mean of 4.7 years (SD: 2.5, range: 1.8–7.8, median: 4.0) as compared to 3.4 years (SD: 2.2, range: 1.0–8.1, median: 3.1; *p* = 0.463) in the control group (Table 4), but this was not statistically significant.

**Figure 1:**
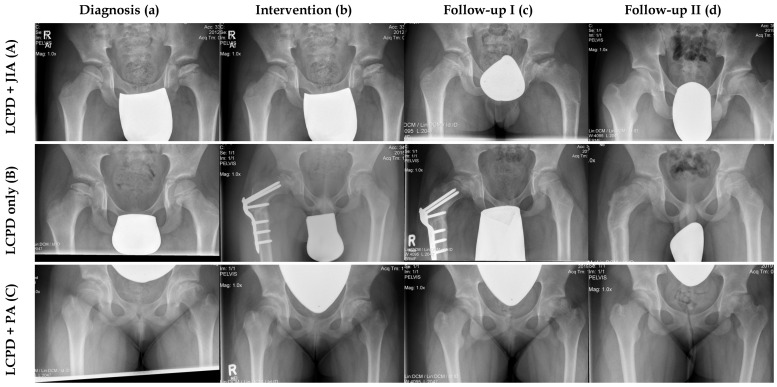
Comparison of three radiological courses between one patient with LCPD only and two patients with concomitant JIA. (**A**) Male patient (Nr. 6) with LCPD and JIA. (**a**) LCPD was diagnosed at the age of 8.7 years (Elizabethtown classification: Ib) and JIA was detected one month later. (**b**) This patient had proximal femoral varus osteotomy (Elizabethtown classification: IIb). (**c**) Anteroposterior radiograph of the pelvis after implant removal (Elizabethtown classification: IIIa). (**d**) This patient had a very good clinical and radiological outcome at the last follow-up (FU: 7.9 years, mHHS: 95, Stulberg: II). (**B**) Male patient with LCPD only (Nr. 9, control group). (**a**) LCPD was diagnosed at the age of 7.9 years in this patient (Elizabethtown classification: Ib). (**b**) He underwent proximal femoral varus osteotomy and permanent trochanteric epiphysiodesis (Elizabethtown classification: IIb). (**c**) Anteroposterior radiograph of the pelvis during the course of disease (Elizabethtown classification: IIIb). (**d**) This patient had a very good clinical and radiological outcome at the last follow-up (FU: 4.9 years, mHHS: 100, Stulberg: II). (**C**) Female patient with LCPD and psoriatic arthritis (Nr. 8). (**a**) LCPD was diagnosed at the age of 11.8 years (Elizabethtown classification: Ib), followed by psoriatic arthritis 2 months later. (**b**) This patient underwent retrograde drilling of the left femoral head (Elizabethtown classification: IIb). (**c**) Anteroposterior radiograph of the pelvis during the course of disease (Eliizabethtown classification: IIIb). (**d**) At the last follow-up the patient had a poor clinical and radiological outcome (FU: 2.8 years, mHHS: 57, Stulberg: V).

With regard to fragmentation severity, the lateral pillar classification was applied in seven JIA hips and ten control hips. A reduction of less than 50% of the lateral pillar height (Herring B) was seen in three JIA patients and in four controls. Severe collapse of the lateral pillar (>50% of the height, Herring C) was noted in four JIA patients and in six controls. None of the patients had a normal height of the lateral pillar of the femoral head. At latest follow-up, eight hips of the JIA group and eight control hips were classified according to the Stulberg classification. Five JIA hips and five controls were classified as Stulberg I/II, two hips in each group were classified as Stulberg III, and one hip of the JIA group as well as one control hip were classified as Stulberg V (Figure 1). Two control hips were not assessed, as they did not reach stage IV (complete healing/remodeling) according to the modified Elizabethtown classification during follow-up.

Radiographic acetabular values were assessed on AP radiographs of the pelvis in all affected and unaffected hips (36 hips analyzed) at the last follow-up. The results are summarized in Table 5.

Hip motion was limited during the course of disease, especially in the JIA group (Table 6). Patients had a reduced mobility in both hips (the LCPD side was more affected), except for extension and flexion. Hip rotation was reduced in the JIA group as compared to controls throughout the study period (both hips), although the difference was only significant for one year. Internal rotation of the hip was painful in most of the JIA patients (6/8) as compared to the control group (3/10; *p* = 0.077).

The modified Harris Hip Score (mHHS) was assessed in all patients of both groups at the last follow-up. Four children with JIA and all control patients had an excellent result, one JIA patient had a good result, one JIA patient had a fair result, and two patients with JIA had poor results, according to the mHHS score. The mean Harris Hip Score was lower in the JIA group (mean: 84.3, SD: 16.4, range: 57–100, median: 91) as compared to controls (mean: 98.4, SD: 2.1, range 95–100, median: 99.5; *p* = 0.021). Half of the JIA patients (4/8) had a mean leg length inequality of 10 mm (SD: 4, range: 5–15, median: 10), with the LCPD-affected leg being shortest at the last follow-up. In the control group, a leg length discrepancy (LLD) in disfavor of the LCPD-side was observed in three out of ten patients at the latest follow-up (mean 10, SD: 9, range: 5–20, median: 5; *p* = 1.000).

At the most recent follow up, three out of eight patients with JIA had no active rheumatic disease, and four JIA patients were still receiving medication. Three children of the JIA group and two controls had a persistent limping gait at the last follow-up. Six JIA patients and four controls reported reduced sport activity. Crutches were used by one JIA patient for walking. However, full weight-bearing was unrestricted in both groups at the latest follow-up.

## 4. Discussion

JIA in combination with LCPD is extremely rare. JIA represents the most common rheumatic disease in children, with a reported incidence of 1.9 to 23 per 100,000 and an onset before 16 years of age [3,6,12]. The peak incidence of LCPD is between 4 and 8 years of age, with 5.1 to 11.6 affected children per 100,000 per year [8]. This disease occurs more frequently in boys, while girls are more often affected by JIA [24]. Most of the children included in this study were boys (7/8 in the JIA group and 8/10 in controls). To our knowledge, this is the first report that describes the course and outcome of LCPD in a series of children with a verified diagnosis of JIA.

Oligoarticular subtype is the most frequent type of JIA, and it was the predominant JIA subtype in this study (5/8). The gender distribution and the slightly higher average age than usual of this group may have occurred randomly. Most of the JIA patients had a positive family history of rheumatic or chronic inflammatory disease as compared to the control group (5/8 versus 2/10). This is consistent with previously published data [3]. Although elevated inflammatory and rheumatic markers were observed in five out of eight JIA patients (1/6 in the control group), previous studies have demonstrated that this is not indicative for JIA [5,25]. Routine laboratory studies should include serum vitamin D levels, as vitamin D deficiency was found in most of the JIA patients (6/8) in this series, while controls were rarely affected (1/10). Vitamin D has been implicated in the pathogenesis of autoimmune disorders and plays an important role in the maintenance of mineral homeostasis and bone health [26,27]. Shevchenko et al. reported that vitamin D deficiency is associated with increased disease activity and a higher risk for the development of uveitis in their series of 69 JIA patients (15 healthy children served as controls) [28]. It has been proven that a reduced level of outdoor physical activity correlates with Vitamin D deficiency as well [29,30]. We believe that physical inactivity due to complaints involving joints may have also contributed to vitamin D deficiency and obesity in children with JIA in our study.

Joseph et al. [16] reviewed 2634 pairs of radiographs (anteroposterior and lateral) of 610 patients with LCPD and divided the disease into seven stages (modified Elizabethtown classification). The initial four stages of the disease (Ia to IIb) lasted approximately 3.5 to 4 months each [16]. In the study by Herring et al., the length of the fragmentation phase (IIa and IIb) was reported to be 9 months [31]. Marklund and Tillberg found that the duration of the reparative phase (IIIa and IIIb) was greater than the sum of the duration of stages Ia to IIb [32]. Given the limitations of estimating the duration of the different stages by analyzing sequential radiographs, we found no differences between the JIA group and controls. Although the transition time from stage II to IV was longer in JIA patients, with a mean of 4.7 years as compared to 3.4 years in the control group (not significant), the mean duration of the critical stages I and II was similar in both groups. The mean duration of stage III was greater than the sum of the duration of stages I and II in both of our groups, which is consistent with the findings of Marklund and Tillberg [32].

Patients underwent a variety of surgical treatments for LCPD in our study (except for one patient in each group), with the proximal femoral varus osteotomy being the most common surgical procedure in both groups. In a prospective multicenter study, Herring et al. reported that in the lateral pillar B group and B/C border group, the outcomes of surgical treatment (femoral or pelvic osteotomy) were significantly better than those of nonoperative treatment in children over the age of 8.0 years at the onset of LCPD using the Stulberg classification [31]. They found no difference between operative and nonoperative treatment in patients 6.0 to 8.0 years of age in this group. In the Norwegian prospective nationwide study on LCPD, the authors found that in lateral pillar group C hips, better outcomes occurred (Stulberg classification) after proximal femoral varus osteotomy in children 6.0 to 7.9 years of age, as compared to controls (nonoperative treatment) [33]. The authors observed no differences in femoral head sphericity between the femoral osteotomy and control group in lateral pillar group B hips. The degree of varus angulation was 20 in each JIA patient and 19.4 (range 10–30) in controls in our study. This is consistent with previous reports, as the mean degree of varisation was 23 (range 15–34) in the series of Terjesen et al., for instance [33].

With regard to fragmentation severity, we found no differences between the JIA group and controls using the original lateral pillar classification. This has been proven to be of prognostic value in LCPD [18,19,20]. The Stulberg classification, as a predictor of long-term outcome in LCPD, was applied at the latest follow-up. We observed no differences in femoral head sphericity between the JIA group and controls, with five hips being classified as Stulberg I/II (good result), two hips as Stulberg III (fair result), and one hip as Stulberg V (bad result) in each group. Stulberg V hips tend to develop osteoarthritis early [7,20,31,32,34,35].

Radiographic acetabular values were assessed at the last follow-up in all hips. The acetabular index, which is commonly used in the diagnosis of adult hip dysplasia, was normal in affected as well as unaffected hips of both groups according to Tannast et al. [22]. The mean acetabular index did not differ between the affected hips of the JIA group and controls. The sharp angle is a reliable measurement in skeletally immature children and in skeletally mature patients with developmental dysplasia of the hip (DDH) [36,37]. It has been also validated in children with LCPD by Huhnstock et al. [36]. The authors reported that the mean value of the Sharp’s angle was 45° at LCPD diagnosis and remained stable at the 5-year follow-up, while the mean Sharp’s angle decreased from 45° to 42° in unaffected hips during that time [36]. Consistent with these results, the mean Sharp’s angle was between 40° to 44° in affected as well as in unaffected hips of both groups in our study. It did not differ between the affected hips of the JIA group and controls. The extrusion index was normal in affected hips of the JIA group but was increased in affected hips of the controls. This difference was significant, but there was one outlier in the control group, which might have influenced the results. Considering all measured acetabular values, there was no clear evidence for hip dysplasia or severe overcoverage in the JIA group and controls.

Clinical evaluation revealed some differences between both groups in our study. Hip extension, flexion, and abduction did not differ between the JIA group and controls during follow-up. Hip rotation was reduced in both hips of the JIA group as compared to controls throughout the study period, but the difference was significant for one year only. At the last follow-up, hip rotation did not differ between both groups. Reduced hip mobility may be due to the fact that hip involvement tends to be bilateral and develops in 30–50% of children with JIA [1]. The mHHS was assessed in all patients at the last follow-up with an excellent result in four patients, a good result in one patient, a fair result in one patient, and a poor result in two patients. This score was significantly lower in JIA patients as compared to controls (mean: 84.3 vs. 98.4; *p* = 0.021). The frequency and degree of leg length discrepancy was similar in both of our groups. Four JIA patients and three controls had a mean leg length inequality of 10 mm each, with the LCPD-affected leg being the shortest at the last follow-up. This is consistent with previous studies focusing on children with LCPD [34]. The radiological outcome of LCPD did not differ between both groups in this study, and the clinical outcome was only slightly better in controls, although patients of the JIA group were an average of 2 years older on LCPD diagnosis. We know that young children, especially children under the age of 6 years at LCPD diagnosis, have a better outcome than those aged six and older [34,38].

A more robust way of demonstrating that the course of LCPD is not appreciably altered by concomitant JIA is a comparison with the previous study of Rupprecht et al [39]. serving as another control group. This study was conducted at our institution as well. They analyzed the results in a large single-center cohort of 52 children affected by LCPD who underwent combined pelvic and femoral osteotomies.

The mean age at diagnosis was 6.9 years, which is slightly younger than in our JIA cohort. Based on the radiographic evaluation by Joseph, both cohorts deviate from each other [16]. A total of 21% of the hips of the combined osteotomy group and 62.5% of our JIA group were classified as being in the late initial phase (Ib), 40% versus 25% were assigned to the early fragmentation phase (IIa), 31% of the combined osteotomy group hips presented at the late fragmentation stage (IIb), and 8% versus 12.5% were assigned to the reossification phase (IIIa/b).

After a mean follow-up of 10.8 years, they noted satisfactory clinical results in 87% and radiographic results in 52% of cases, which is comparable to our JIA group. The reported Harris hip score by Rupprecht et al. was 90 ± 13.2. Apart from one outlier (mHHS: 57), our results compare favorably, with a mean mHHS of 84.3 ± 16.4.

Rupprecht et al. classified 52% of their patients as having a Stulberg class-I or -II hip; 29% a class-III hip; 12% a class-IV hip; and 8% a class-V hip. These findings are similar to our results (62.5% had Stulberg class-I or -II, 25% class-III and 12.5% class-V hips). The percentage of patients with a reduction of less than 50% of the lateral pillar height (Herring B; 32% vs. 43%) or a severe collapse (Herring C; 68% vs. 57%) do not differ between the double osteotomy patients and our JIA children.

Taken together, Rupprecht et al. did not find better clinical and radiographic outcomes in children with LCPD after combined pelvic and femoral osteotomies as compared to our JIA group, indicating that there is no evidence that JIA adversely affects the course of LCPD. This may be due to the fact that JIA was diagnosed simultaneously with LCPD in seven of eight children (within 4 months) and that they were all evaluated and treated (anti-rheumatic medication) by experienced local pediatric rheumatologists immediately and throughout the course of the disease. Appropriate treatment by pediatric rheumatologists may have contributed to the outcome of LCPD in this study. Physicians should be aware that children with LCPD may have JIA, too. In particular, if other joints are affected, in the case of a positive family history of rheumatic or chronic inflammatory disease, or in the case of a suspicious patient history (varying joint complaints), further investigation is recommended (e.g., magnetic resonance imaging), and patients should be referred to a pediatric rheumatologist early.

The limitations of this study include the retrospective design and the limited number of patients. The small sample size resulted from our focus on a complete clinical and radiological documented long-term outcome. A selection bias may have occurred due to the fact that children with milder forms/courses of Perthes disease are usually treated by established orthopedists in their outpatient clinic and were not referred to our specialized pediatric orthopedic center. Unfortunately, the mHHS as a parameter for clinical outcome was only assessed at the end of treatment in each patient. However, we believe that this study is of clinical importance and provides useful findings that can serve as a benchmark for future studies (more included patients, prospective design), which are needed before reliable conclusions can be drawn.

## Figures and Tables

**Table 1:** LCPD patients with a verified diagnosis of juvenile idiopathic arthritis.

Patient Number	Type of JIA	Sex	Age at Onset LCPD (y)	Age at Onset JIA (y)	Lateral Pillar Classification	Stulberg Classification	mHHS (p)	Treatment	Follow-Up Time (y)
1	Oligoarticular	m	7.1	7.0	C	II	87	Arthrography, proximal femoral varus osteotomy, Salter osteotomy	11.1
2	Enthesitis-related arthritis	m	9.1	9.2	-	III	100	Permanent trochanteric epiphysiodesis	12.4
3	Oligoarticular	m	14.7	14.6	B	III	76	Distraction frame, retrograde drilling, open waisting of femoral neck, open arthrolysis and partial arthroscopic synovialectomy	10.5
4	Oligoarticular	m	5.0	4.6	C	II	65	Arthrography, proximal femoral varus osteotomy, permanent trochanteric epiphysiodesis	3.4
5	Enthesitis-related arthritis	m	3.0	11.5	C	I	98	Proximal femoral varus osteotomy	18.3
6	Oligoarticular	m	8.7	8.8	B	II	95	Arthrography, proximal femoral varus osteotomy	7.9
7	Oligoarticular	m	5.2	5.2	B	I	96	Nonoperative treatment	6.8
8	Psoriatic arthritis	f	11.8	12.0	C	V	57	Hip joint puncture, retrograde drilling	2.8

LCPD = Legg-Calvé-Perthes disease, JIA = juvenile idiopathic arthritis, m = male, f = female, y = years, p = points, - = no data available.

**Table 2:** Patients with LCPD only (control group).

Patient Number	Sex	Age at Onset LCPD (y)	Lateral Pillar Classification	Stulberg Classification	mHHS (p)	Treatment	Follow-Up Time (y)
1	m	7.2	C	II	100	Arthrography, permanent trochanteric epiphysiodesis, proximal femoral varus osteotomy	4.2
2	m	2.9	C	V	95	Proximal femoral valgus and derotation osteotomy	11.4
3	m	10.0	C	III	98	Arthrography, permanent trochanteric epiphysiodesis, proximal femoral varus osteotomy	3.5
4	m	9.8	B	II	97	Arthrography, distraction frame, permanent trochanteric epiphysiodesis, proximal femoral varus osteotomy	8.7
5	m	3.7	C	-	100	Arthrography, proximal femoral varus osteotomy, Salter’s pelvic osteotomy	7.0
6	m	3.7	B	-	100	Nonoperative treatment	9.0
7	f	5.0	B	III	95	Proximal femoral varus osteotomy, Salter’s pelvic osteotomy, proximal femoral valgus osteotomy	14.4
8	m	3.2	C	II	99	Arthrography, proximal femoral varus osteotomy	3.3
9	m	7.9	B	II	100	Arthrography, permanent trochanteric epiphysiodesis, proximal femoral varus osteotomy	4.9
10	f	7.3	C	II	100	Arthrography, permanent trochanteric epiphysiodesis, proximal femoral varus osteotomy	5.0

LCPD = Legg-Calvé-Perthes disease, m = male, f = female, y = years, p = points, - = no data available.

**Table 3:** Body Mass Index at LCPD diagnosis and at last follow-up.

Classification (Percentiles)	JIA LCPD (D)	Control LCPD (D)	*p* Value	JIA LCPD (FU)	Control LCPD (FU)	*p* Value
Healthy weight (5% to 84%)	42.8	100	* 0.015	62.5	87.5	0.162
Overweight (85% to 94%)	28.6	0	* 0.015	12.5	12.5	0.162
Obesity (95% to 100%)	28.6	0	* 0.015	25	0	0.162
Total	19.8 [19.1] ± 4.2 (15.3–27.5)	16.5 [16.7] ± 1.2 (13.8–17.8)	0.438	23.1 [22.8] ± 7.0 (14.1–37.3)	17.3 [17.3] ± 1.8 (14.9–21.1)	0.467

Values are expressed as frequency in percent, total data as mean ± SD and range in parentheses. [= median]. BMI = Body Mass Index, D = at LCPD diagnosis, FU = at last follow-up, * significant.

**Table 4:** Duration of the different stages of the Elizabethtown classification for the two study groups.

Stages	JIA LCPD	Control LCPD	*p* Value
I	0.5 [0.5] ± 0.2 (0.3–0.8)	0.4 [0.4] ± 0.2 (0.1–0.8)	0.429
II	1.2 [1.3] ± 0.3 (0.8–1.6)	1.2 [0.9] ± 1.0 (0.1–3.6)	0.270
III	3.4 [2.7] ± 2.3 (0.5–6.8)	2.3 [2.5] ± 1.2 (0.9–4.4)	0.505

Values are expressed as mean ± SD and range in parentheses. [= median]. LCPD = Legg-Calvé-Perthes disease, JIA = juvenile idiopathic arthritis. Age in years.

**Table 5:** Radiographic acetabular values at the last follow-up.

Parameter	D	C	OC	SOC	JIA LCPD-Side	Control LCPD-Side	*p* Value	JIA Non-Affected Side	Control Non-Affected Side	*p* Value
Lateral center-edge angle	<22	23–33	34–39	>40	30 [31] ± 7 (17–41)	18 [20] ± 6 (10–25)	* 0.001	34 [33] ± 8 (24–45)	26 [24] ± 5 (19–33)	* 0.016
Medial center-edge angle	>45	35–44	34–29	<28	42 [42] ± 9 (31–58)	46 [43] ± 8 (37–56)	0.408	38 [37] ± 6 (29–50)	42 [42] ± 2 (39–44)	0.068
Acetabular arc	<60	61–65	66–69	>69	73 [74] ± 9 (57–88)	65 [66] ± 6 (50–73)	* 0.043	71 [69] ± 9 (64–92)	68 [67] ± 5 (58–75)	0.460
Acetabular index	>14	3–13	(−7)–2	< -8	8 [10] ± 7 (−5–16)	11 [11] ± 4 (3–16)	0.408	5 [4] ± 6 (-7–12)	9 [11] ± 3 (5–13)	* 0.034
Sharp angle	>43	38–42	34–37	<34	40 [40] ± 4 (34–48)	44 [43] ± 3 (40–52)	* 0.027	40 [40] ± 4 (35–47)	43 [42] ± 3 (39–51)	0.055
Extrusion index	>27	17–26	12–16	<11	24 [22] ± 9 (11–40)	39 [40] ± 15 (21–64)	* 0.038	17 [18] ± 8 (0–26)	27 [25] ± 7 (15–39)	* 0.016

Values are expressed as mean ± SD and range in parentheses. [= median]. Reference values according to Tannast et al [22]: LCPD = Legg-Calvé-Perthes disease, JIA = juvenile idiopathic arthritis, D = dysplasia, C = control, OC = overcoverage, SOC = severe OC, * significant.

**Table 6:** Motion in the course of treatment for the two study groups.

Parameter	JIA LCPD	Control LCPD	*p* Value
**Internal Rotation LCPD-Side**			
First year	19.6 [20] ± 12.3 (6.3–37.3)	23.1 [24] ± 7.4 (10.0–34.2)	0.230
Second year	23.9 [15] ± 18.9 (7.5–60.0)	31.5 [28] ± 9.5 (20.0–45.0)	0.470
Third year	22.0 [25] ± 17.2 (0.0–46.7)	41.1 [40] ± 10.8 (20.0–50.0)	0.067
Last follow-up	26.9 [25] ± 12.2 (10.0–40.0)	27.5 [25] ± 17.8 (0.0–60.0)	1.000
**Internal Rotation Non-Affected Side**			
First year	36.8 [35] ± 14.0 (17.5–55.0)	43.9 [50] ± 10.1 (27.5–53.3)	0.524
Second year	42.7 [40] ± 13.6 (23.3–60.0)	44.6 [41] ± 13.5 (30.0–70.0)	1.000
Third year	35.3 [30] ± 14.3 (20.0–55.0)	46.3 [50] ± 11.1 (30.0–55.0)	0.286
Last follow-up	26.0 [25] ± 13.4 (20.0–50.0)	41.0 [40] ± 12.4 (30.0–60.0)	0.067
**External Rotation LCPD-Side**			
First year	35.3 [34] ± 5.1 (30.0–45.0)	30.1 [31] ± 11.0 (16.0–49.3)	0.181
Second year	42.5 [43] ± 13.0 (30.0–60.0)	34.4 [30] ± 16.4 (12.5–60.0)	0.364
Third year	27.1 [29] ± 12.9 (10.0–40.0)	53.3 [58] ± 8.8 (40.0–60.0)	* 0.010
Last follow-up	38.8 [35] ± 27.0 (10.0–80.0)	46.0 [50] ± 21.7 (0.0–80.0)	0.524
**External Rotation Non-Affected Side**			
First year	47.1 [50] ± 8.6 (35.0–47.8)	55.6 [50] ± 9.0 (45.0–67.5)	0.202
Second year	53.3 [55] ± 2.9 (50.0–55.0)	52.1 [55] ± 15.8 (30.0–70.0)	1.000
Third year	53.4 [53] ± 4.7 (50.0–56.7)	60.0 [60] ± 0.0 (60.0)	0.333
Last follow-up	47.0 [50] ± 21.7 (20.0–80.0)	64.0 [60] ± 11.4 (50.0–80.0)	0.159
**Abduction LCPD Side**			
First year	35.6 [35] ± 10.9 (20.0–50.0)	30.5 [30] ± 9.8 (17.5–50.0)	0.298
Second year	25.0 [30] ± 8.7 (10.0–30.0)	33.7 [38] ± 10.4 (17.5–45.0)	0.190
Third year	30.8 [30] ± 1.7 (30.0–33.3)	37.5 [40] ± 7.6 (25.0–50.0)	0.109
Last follow-up	41.3 [41] ± 5.8 (30.0–50.0)	39.0 [40] ± 13.9 (15.0–60.0)	1.000

Values are expressed as mean ± SD and range in parentheses. [= median]. LCPD = Legg-Calvé-Perthes disease, JIA = juvenile idiopathic arthritis, * significant. Motion in degrees.

## Data Availability

The data presented in this study are available in Table 1, Table 2, Table 3, Table 4, Table 5 and Table 6 and on request from the corresponding author.
